# Analysis of the Sensitivity of K-Type Molecular Sieve-Deposited MWNTs for the Detection of SF_6_ Decomposition Gases under Partial Discharge

**DOI:** 10.3390/s151128367

**Published:** 2015-11-11

**Authors:** Xiaoxing Zhang, Xin Li, Chenchen Luo, Xingchen Dong, Lei Zhou

**Affiliations:** 1 State Key Laboratory of Power Transmission Equipment & System Security and New Technology, Chongqing University, Chongqing 400044, China; E-Mails: cqlixin@cqu.edu.cn (X.L.); 20141113009t@cqu.edu.cn (X.D.); 20131113102@cqu.edu.cn (L.Z.); 2 School of Electrical Engineering, Wuhan University, Wuhan 430072, China; 3 Maintenance Company, State Grid Zhejiang Electric Power Company, Hangzhou 311232, China; E-Mail: luocc89@126.com

**Keywords:** partial discharge, SF_6_ decomposition component, multi-walled carbon nanotube, K molecular sieve, gas sensitivity

## Abstract

Sulfur hexafluoride (SF_6_) is widely utilized in gas-insulated switchgear (GIS). However, part of SF_6_ decomposes into different components under partial discharge (PD) conditions. Previous research has shown that the gas responses of intrinsic and 4 Å-type molecular sieve-deposited multi-wall carbon nanotubes (MWNTs) to SOF_2_ and SO_2_F_2_, two important decomposition components of SF_6_, are not obvious. In this study, a K-type molecular sieve-deposited MWNTs sensor was developed. Its gas response characteristics and the influence of the mixture ratios of gases on the gas-sensing properties were studied. The results showed that, for sensors with gas mixture ratios of 5:1, 10:1, and 20:1, the resistance change rate increased by nearly 13.0% after SOF_2_ adsorption, almost 10 times that of MWNTs sensors, while the sensors’ resistance change rate with a mixture ratio of 10:1 reached 17.3% after SO_2_F_2_ adsorption, nearly nine times that of intrinsic MWNT sensors. Besides, a good linear relationship was observed between concentration of decomposition components and the resistance change rate of sensors.

## 1. Introduction

Sulfur hexafluoride (SF_6_), a type of gas with high stability under normal temperature and pressure conditions, is colorless, odorless, non-toxic, and nonflammable. SF_6_ has become the best electric insulation and arc-quenching medium because of its excellent properties, and for this reason it is widely utilized in the electric power industry [1,2,3,4]. However, under the effect of partial discharge (PD) conditions, SF_6_ gas inside a gas-insulated switchgear (GIS) decomposes and reacts with infiltrating traces of O_2_ and H_2_O and several stable gas byproducts, such as SOF_2_, SO_2_F_2_, SO_2_, and H_2_S, are then generated [5,6]. These components are not only difficult to mix with SF_6_ but they also degrade the insulation performance of SF_6_. Several decomposition components, such as HF and SO_2_, can corrode solid insulating materials and metal parts of the equipment. Such corrosion accelerates insulation aging and ultimately leads to sudden failures in GIS.

Numerous GIS internal insulation defects of different types and degrees have been reported [1,2,7]. Research has shown that under the effect of PD caused by different types of defects, SF_6_ decomposition components vary in type, content, and rate of formation. The research results so far have shown that SO_2_, H_2_S, SOF_2_ and SO_2_F_2_ are the four kinds of gases that are detected to possess the largest concentration in the mixed gases resulting from SF_6_ decomposition, and to some extent the four gases can be considered the characteristic decomposition components [8,9,10,11,12]_._ Based on the fact that the contents of SO_2_, H_2_S, SOF_2_ and SO_2_F_2_ differ largely under different insulation failure conditions, different methods of detecting the four kind of gases have been developed, in order to determine the type of PD inside GIS [13,14].

Among several currently available methods to detect the components of SF_6_ gas decomposition products, the gas sensor method has the advantages of fast detection speed, high efficiency, and easy access to achieve online monitoring. Gas sensors made of carbon nanotubes (CNTs) possess excellent properties, such as high sensitivity, low energy consumption, fast response, small size, and the ability to operate at room temperature [15,16]. Extensive research on the detection of SF_6_ decomposition components through the use of carbon nanotube gas sensors is currently being conducted. The detection of SO_2_ and H_2_S, two types of important decomposition components of SF_6_, has been thoroughly investigated. However, studies on the detection of two other types of components, namely, SOF_2_ and SO_2_F_2_, are rare, and no breakthrough progress has been reported [17,18,19,20,21,22,23].

The gas-sensing characteristics of intrinsic carbon nanotube sensors can be improved through deposition of different types and concentrations of molecular sieves. In [23] the authors adopted the 4 Å molecular sieve deposition method and performed gas sensing experiments. The results showed that carbon nanotubes of different concentrations exhibit good sensitivity and selectivity to SO_2_ and H_2_S. However, in the current study, the 4 Å molecular sieve-deposited carbon nanotube sensors exhibited almost no sensitivity to SOF_2_ and SO_2_F_2_. Hence, we propose a K-type molecular sieve deposition method to modify multi-walled carbon nanotubes (MWNTs) to achieve improved sensitivity and selectivity, which could lay the foundation for the development of sensors for online detection of SF_6_ decomposition components.

## 2. Experimental Section

### 2.1. Structure and Characteristics of K-Type Molecular Sieve

**Figure 1:**
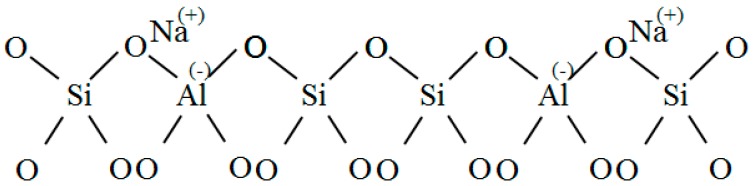
Tetrahedral framework structure.

KDHF-03 zeolite (K-type molecular sieve) is a synthetic and modified molecule sieve used for high-voltage electrical equipment. Its main components are 13X zeolite, 5 Å zeolite and clay. Its chemical formula is Al_2_O_3_·4SiO_2_·xFe_2_O_3_·yMgO·nH_2_O. A molecular sieve is essentially a type of aluminosilicate of which the basic structures are silicon–oxygen tetrahedra and aluminum-oxygen tetrahedra (Figure 1). Two adjacent silicon-oxygen (or aluminum-oxygen) tetrahedra constitute the chamfered octahedral (β cage) by sharing an oxygen atom.

**Figure 2:**
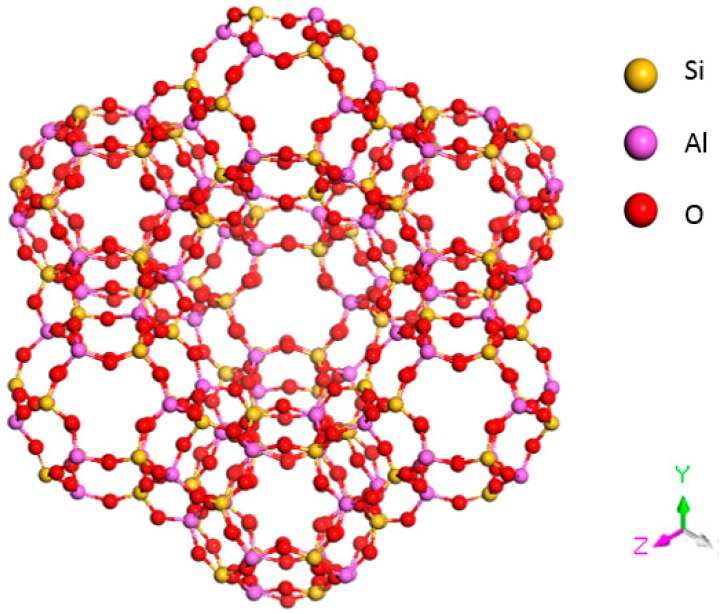
Structural model of an X-type molecular sieve.

X zeolite is composed of cubic crystals; the mole ratio of SiO_2_/A1_2_O_3_ in X zeolite is approximately 2.2 to 3.0. The ideal cell of X zeolite is Na_86_(Al_86_Si_106_O_384_)·264H_2_O. Each structural unit is composed of eight β cages arranged in a diamond crystal pattern. Adjacent β cages are interconnected by six rings and oxygen bridges. The structure of an X-type molecular sieve is shown in Figure 2.

**Figure 3:**
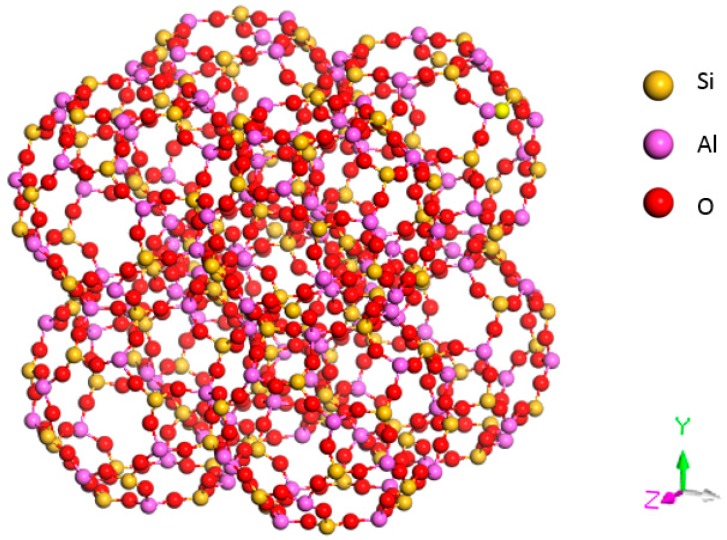
Structural model of an A-type molecular sieve.

The structure of 4 Å zeolite is similar to that of a NaCl crystal (see Figure 3). Each vertex of the cube has a β cage, and two adjacent β cages are interconnected by four rings through an oxygen bond. Eight β cages connect to form a large α cage in the center.

As shown in Figure 1, aluminum is trivalent, and four oxygen atoms surround an aluminum atom in the tetrahedral framework structure of zeolite. The aluminum-oxygen tetrahedron has a negative charge, and the valence electron of an oxygen atom is not neutralized. To maintain charge balance, cations (usually an alkali metal or alkaline earth metal ion) neutralize the negative charge. The metal cations in 4 Å zeolite, which obtained its name because of its effective aperture of 4 Å, are sodium ions. A 5 Å zeolite is obtained by replacing more than 70% of the Na^+^ with Ca^2+^ in a 4 Å zeolite based on zeolites’ ion exchange properties. The metal cations in a 5 Å molecular sieve are Ca^2+^ and Na^+^. These metal cations and oxygen atoms of the molecular sieve’s skeleton constitute a relatively stable structure. Therefore, such particular zeolite structure possesses a large surface area and a strong surface field. In the tetrahedron, that metal cations and anion skeleton formg dipoles makes it easier for strong polar gases to be adsorbed.

### 2.2. Preparation of K-Type Molecular Sieve-Deposited MWNT Thin-Film Sensors

The MWNTs in this study are made of black powder, which was purchased from the Chinese Academy of Sciences Chengdu Institute of Organic Chemistry and prepared through chemical vapor deposition. The parameters of the MWNTs are listed in Table 1.

**Table 1:** Parameters of MWNTs.

	Diameter (nm)	Length (μm)	Surface area (m^2^/g)	Purity (wt%)	Residual Catalyst (wt%)
MWNTs	20–30	10–30	>100	>95	<1.5

KDHF-03 molecular sieves, of which the parameters are shown in Table 2, were purchased from Shanxi Yixiu Co., Ltd. (Xi’an, China).

**Table 2:** Technique specification of K molecules.

	Shape	Granularity	Surface Area (m^2^/g)	Effective Substance Contents (wt%)	Packing Density (g/mL)
K zeolite	globular	3–5 mm	500 ± 30	>99.99	0.68

The MWNTs and molecular sieves were weighed, and different mass ratios of 1:1, 3:1, 5:1, 10:1, and 20:1 were selected. It should be noted that, at different mass ratios, the mass of MWNTs was measured at a certain value of 1 g, in order to obtain mixed solutions with the same concentration of MWNTs. The materials were then dispersed into anhydrous ethanol to prepare 200 mL of mixed solution. After 90 min of stirring with an ultrasonic oscillator, a uniformly dispersed suspension was obtained. A portion of the supernatant (50 mL) was taken out and dispersed in anhydrous ethanol to again prepare 200 mL of mixed solution. The mixed solution was then dispersed for 60 min using an ultrasonic oscillator.

**Figure 4:**
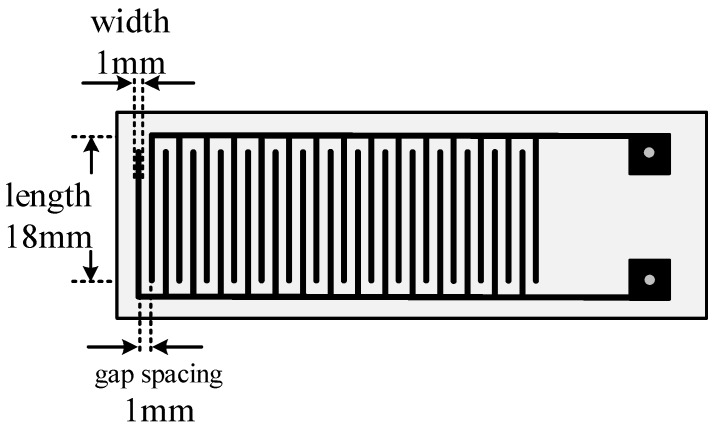
Diagram of the interdigital electrode structure.

The MWNT sensor substrate is made of printed circuit boards. Copper interdigital electrodes with thickness of 30 µm, width of 1 mm and gap spacing of 1 mm are etched on the substrate, shown in Figure 4. A burette was utilized to place a trace of mixture solution on the surface of the interdigital electrodes. The interdigital electrodes were then placed in an oven at 80°C to create a uniform, dense, smooth film. The film served for the detection of characteristics of the decomposed SF_6_ components.

**Figure 5:**
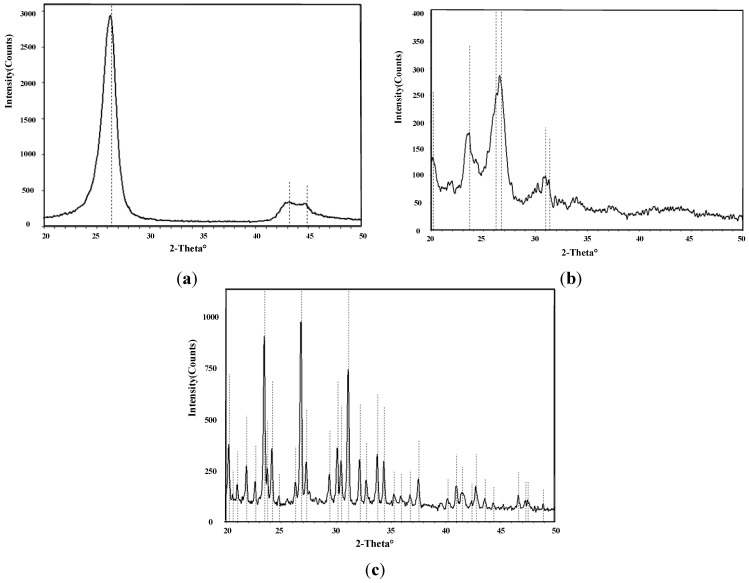
(**a**) XRD of MWNTs; (**b**) XRD of molecular sieve-deposited MWNTs; (**c**) XRD of K-type molecular sieve.

Figure 5 shows X-ray diffraction (XRD) images of intrinsic MWNTs, K-type molecular sieve-deposited MWNTs, and K-type molecular sieve. With 2-Theta ranging from 20° to 50°, there are six characteristic peaks observed in the XRD image of molecular sieve, which appear at the degree of approximately 20°, 22°, 24°, 27°, 31°, and 34°. Correspondingly, the above characteristic peaks at six degrees of approximately 20°, 22°, 24°, 27°, 31°, and 34°, also existed in the XRD spectrum of the mixture of intrinsic MWNTs and K-type molecular sieve. No other obvious characteristic peak is inconsistent between the XRD image of intrinsic MWNTs and XRD image of K-type molecular sieve. This accordance indicates that the K-type molecular sieve was deposited on the MWNTs.

**Figure 6:**
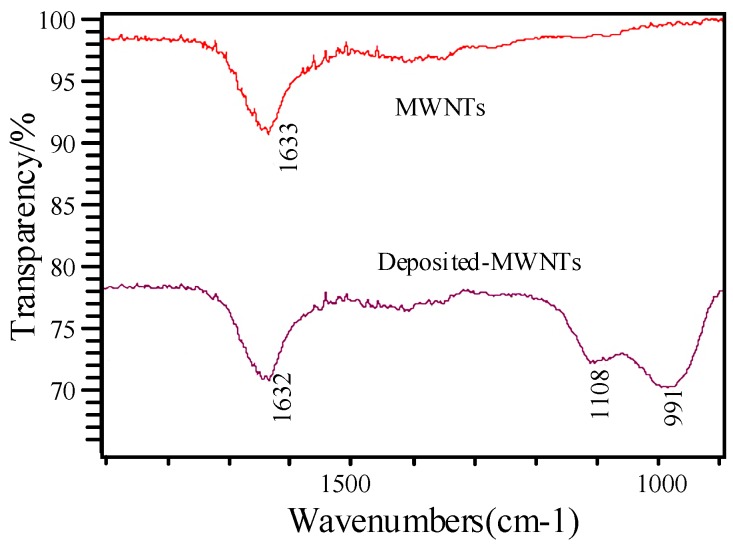
Infrared absorption spectra of MWNTs and deposited MWNTs.

The infrared absorption spectra method was used to provide additional evidence to estimate whether some molecules were mixed with the intrinsic MWNTs. Figure 6 shows a comparison of the intrinsic and K-type molecular sieve-deposited MWNTs; the comparison was conducted with a Nicolet 5DXCFT-IR infrared spectrometer (Thermo Nicolet Corporation, Madison, WI, USA).

The infrared spectra in Figure 6 show an evident absorption peak from 1000 cm^−1^ to 1100 cm^−1^ in the MWNTs deposited with K-type molecular sieve. The reason for this may be the asymmetric stretching vibration of the tetrahedron in the molecular sieve, which can be treated as supporting evidence that proves the K-type molecular sieve was deposited on the MWNTs.

### 2.3. Gas Sensing Test Platform

**Figure 7:**
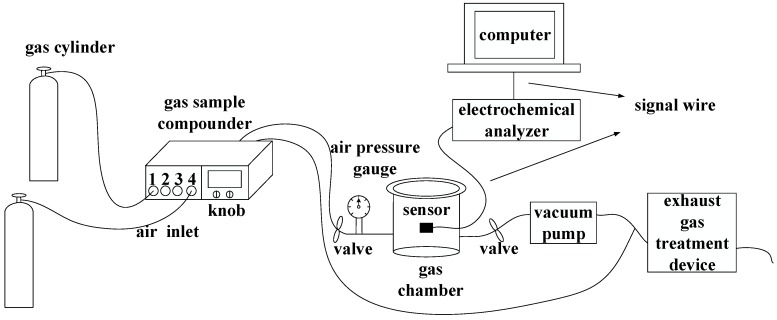
Schematic of the experimental and testing system.

The entire test system includes cylinders, a gas sample compounder, a gas chamber, an air pressure gauge, an inlet/outlet valve, a CHI electrochemical analyzer (Chenhua Instruments Corporation, Shanghai, China), a computer, a vacuum pump, and an exhaust gas treatment device (Figure 7). Two cylinders contain different gases: pure SF_6_ and some SF_6_ decomposition component gases (such as SO_2_, H_2_S, SOF_2_, or SO_2_F_2_) whose carrier gas are SF_6_. A gas sample mixer with four inlets is used to obtain different concentrations of gases. The cylinder of pure gas SF_6_ is connected to the fourth inlet while the cylinder containing SF_6_ decomposition component gases is connected to any one of inlets 1, 2, and 3. What’s more, there are two knobs to allow the gas mixtures to flow either to the gas chamber or to the tail gas treatment device. The air pressure of cylinders should be controlled at 0.2–0.3 MPa to meet the requirements of gas sample mixer.

Gas sensitivity tests were performed in a sealed gas chamber at atmospheric pressure. An air pressure gauge outside the gas chamber was used to measure the pressure of the gas chamber. The flow rate of the gas mixture output was controlled at 0.2 L/min by the gas sample mixer. The conductivity changes of the sensor were detected with a CHI electrochemical analyzer, and test data were recorded on a computer.

**Figure 8:**
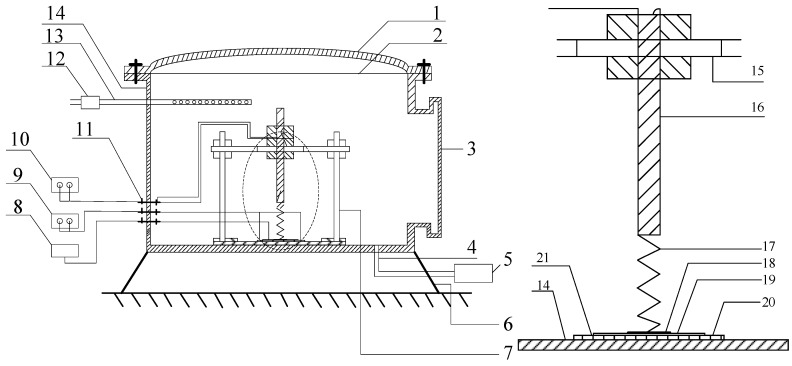
Schematic diagram of the gas chamber.

The gas chamber is approximately cylindrical, with a radius of 100 mm, height of 115 mm, and an inner volume of around 3.6 L, which includes the following parts: (1) a gas chamber cover made of glass; (2) an O-shape seal ring; (3) quartz glass cover; (4) gas outlet; (5) vacuum pump; (6) supporting foot; (7) sensor fixation apparatus; (8) digital thermometer; (9) AC voltage regulator; (10) electrochemical analyzer; (11) wiring terminal; (12) flowmeter which plays a subsidiary function; (13) gas inlet; (14) cylinder block; (15) guideway; (16) electrode; (17) mechanical spring; (18) platinized platinum; (19) sensor; (20) ceramic heating plate; (21) square groove. Figure 8 shows the structure of the gas chamber used in the experiment.

The electrochemical analyzer is a common electrochemical measurement system. The CHI600D series instruments employed in the tests integrates many common electrochemical measurement techniques, including constant voltage, constant current, potential scanning, current scanning, potential step, AC impedance, current voltammetry, and other techniques. The real-time resistance of sensors can be directly obtained according to the output files of the CHI600D electrochemical analyzer which measures the impedance of sensors. By setting the sample time and measurement precision, numerous accurate resistance parameters can be obtained. Moreover, using an electrochemical analyzer to collect data avoids errors caused by the inadequate precision of resistance measuring tools, such as impedance analyzers and human eyes.

### 2.4. Procedures of the Gas-Sensing Test for MWNT Sensors

The experimental steps were as follows:

(1) The test device was checked to confirm that the connections were correct and complete. With the test method of the electrochemical analyzer selected and parameters set, the initial resistance value R_0_ of the MWNT sensor was recorded.

(2) A gas tightness test was carried out with vacuum gauge and outlet valves open, and the inlet valve closed. The vacuum pump was switched on to pump air from the cylinder. The data display on the vacuum gauge was observed. Thereafter, the gas outlet valve and the vacuum pump were turned off after the cylinder was under vacuum. The number displayed on the vacuum gauge was recorded. After 12 h, the number displayed on the vacuum gauge was recorded again and compared with the previous number. No change indicates good airtightness of the chamber. Afterward, the resistance value R_0_’ of the MWNT sensor was recorded and compared with R_0_, which is the resistance value before vacuum pumping. No change indicates that the resistance of the sensor is stable with no zero drift.

(3) The SF_6_ decomposition products were injected into the sealed chamber through an inlet valve. The resistance values (R) of the MWNT sensor were recorded with an electrochemical analyzer when no change was observed.

(4) When the test was completed, N_2_ gas was passed into the chamber to ensure that the sensor regained its initial resistance value. After implementing all the steps, test data recording was stopped, and the results were saved. The above steps were repeated to test the other sensors.

## 3. Results and Discussion

### 3.1. Characteristic Curves of Gas Sensors

The gas sensitivity of K-type molecular sieve-deposited MWNT gas sensors to the SF_6_ gas decomposition products (SOF_2_ and SO_2_F_2_) was tested. Before the experiment, the stability of the gas sensors was tested to ensure that no sensor zero drift occurred. All the response diagrams include the action time of MWNT gas sensors on the horizontal axis (the tested gas was pumped into the gas chamber from 0 s), and the resistance change rate of sensors on the vertical axis.

**Figure 9:**
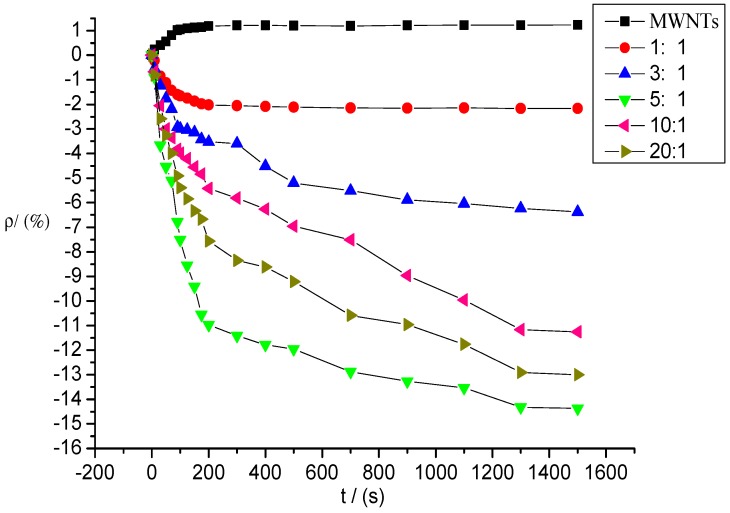
Response of MWNT sensor to SOF_2_ at 100 ppm concentration.

Figure 9 shows the gas response curves of intrinsic and deposited MWNT sensors to SOF_2_ at a concentration of 100 ppm; the mixture ratios of the sensors are 1:1, 3:1, 5:1, 10:1, and 20:1.

As shown in Figure 9, the response curves of the five deposited MWNT sensors began to decline significantly after contact with SOF_2_ gas. The sensor resistance values began to decrease, whereas the resistance values of the intrinsic MWNT sensors increased. The response curves of the sensors gradually stabilized over time before finally reaching a steady value, that is, the maximum resistance change rate mentioned above. Compared with the intrinsic MWNT sensors, the resistance changes in the K-type molecular sieve-deposited MWNT sensors after SOF_2_ adsorption were significantly higher. The curves were steeper, and the resistance values decreased more rapidly.

During the detection of SOF_2_ gas with a concentration of 100 ppm, the resistance of the intrinsic MWNT sensors increased, and the absolute value of resistance change was 1.23%. The absolute values of the gas sensors’ resistance changes produced at different mixture ratios were enhanced at various degrees after SOF_2_ adsorption. The resistance change rates of sensors with mixture ratios of 1:1, 3:1, 5:1, 10:1, and 20:1 were −2.17%, −6.37%, −14.37%, −11.27%, and −13.0%, respectively. The deposited molecular sieve not only improved the sensitivity of MWNTs to SOF_2_ gas (the resistance change rate of sensors increased significantly), but also changed the absolute tendency of the resistance change (the resistance values of the deposited sensors decreased, whereas the resistance values of the intrinsic sensors increased). In the deposited sensors, the resistance change rates of sensors at mixture ratios of 5:1, 10:1, and 20:1 increased to 14.37%, 11.27%, and 13%, respectively, which are 10 times that of intrinsic sensors. The response rate was markedly improved when the adsorption property of the deposited MWNT sensors increased significantly.

**Figure 10:**
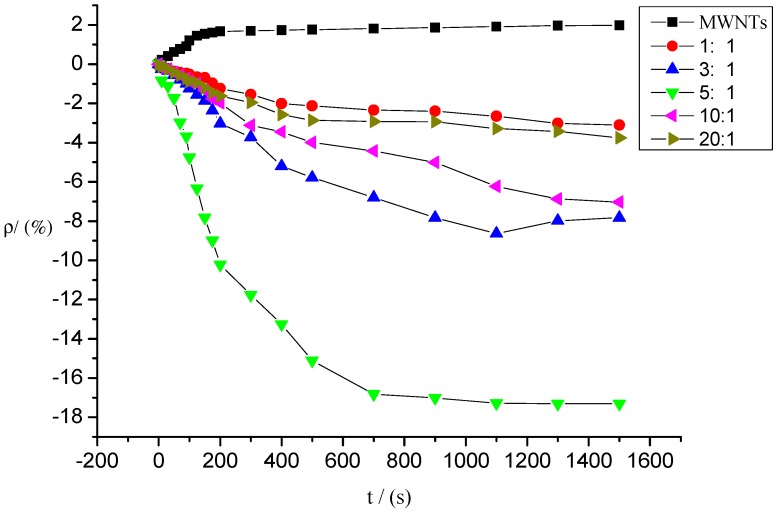
Response of MWNT sensor to SO_2_F_2_ at 100 ppm concentration.

Similarly, the intrinsic MWNT gas sensors and deposited MWNT sensors whose mixture ratios were 1:1, 3:1, 5:1, 10:1, and 20:1 were utilized to detect SO_2_F_2_ gas at a concentration of 100 ppm. Figure 10 shows the gas response curves of these sensors to SO_2_F_2_. Similar the SOF_2_ detection, the gas response curves of the five deposited MWNT sensors began to decline to different degrees after the sensors came into contact with SO_2_F_2_. The resistance values of the intrinsic MWNT sensors increased. The response curves of the sensors stabilized gradually over time and finally reached a stable value.

Compared with the intrinsic MWNT gas sensors, the resistance changes in the MWNT gas sensors deposited with K-type molecular sieve were significantly larger, and the resistance values decreased rapidly. Consequently, the response curve to SO_2_F_2_ was steeper.

During SO_2_F_2_ detection at 100 ppm concentration, the resistance of the intrinsic MWNT sensors increased, and their absolute value changed by 1.98%. The absolute values of gas sensors’ resistance change with different mixture ratios improved to various degrees after SOF_2_ adsorption. The resistance change rates of sensors with mixture ratios of 1:1, 3:1, 5:1, 10:1, and 20:1 were −2.17%, −6.37%, −14.37%, −11.27%, and −13.0%, respectively. Similar to the results in SOF_2_ detection, the molecular sieves mixed with MWNTs improved the sensitivity to SO_2_F_2_, significantly enhanced the sensors’ resistance change rate, and changed the absolute tendency of the resistance change (*i.e.*, the resistance values of the deposited sensors decreased, whereas the resistance values of the intrinsic sensors increased). In the deposited sensors, the resistance change rate of the MWNT sensors with a mixture ratio of 5:1 showed the largest improvement of 17.32%, which was approximately nine times that of the intrinsic sensors. The adsorption ability and response rate of the MWNT sensors to SO_2_F_2_ at a mixture ratio of 10:1 evidently increased.

Figure 9 and Figure 10 show that the response time of deposited carbon nanotubes to SOF_2_ and SO_2_F_2_ was shortened, especially the response time to SO_2_F_2_. In terms of resistance change rate and response time, K-type molecular sieve-deposited MWNTs exhibited better response characteristics to SO_2_F_2_ and SOF_2_ than the intrinsic MWNT gas sensors.

### 3.2. Selectivity of Different Sensors to SOF_2_ and SO_2_F_2_

**Figure 11:**
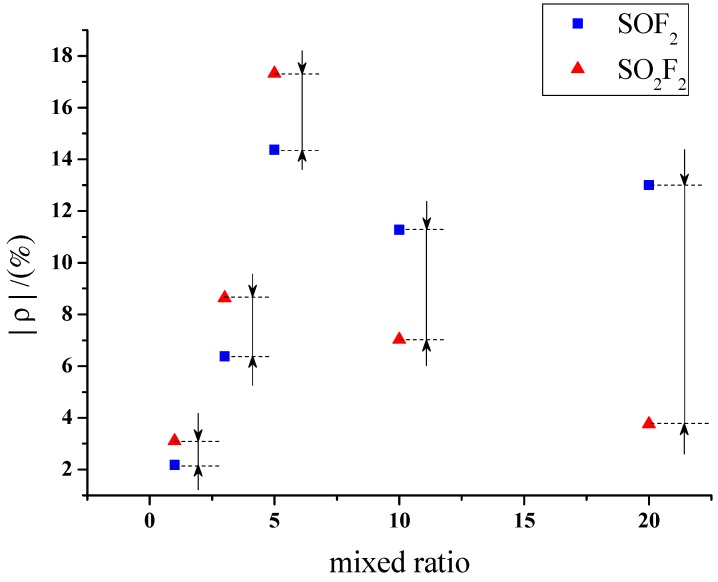
Response of MWNT sensor to SOF_2_ and SO_2_F_2_.

Figure 9 and Figure 10 reveal that large differences exist among the responses of different sensors to SOF_2_ and SO_2_F_2_. The gas-sensing response of sensors with different mixture ratios to SOF_2_ and SO_2_F_2_ is shown in Figure 11. The resistance change rate of the sensor with a mixture ratio of 5:1 was considerably larger than that of the sensor with other mixture ratios after SO_2_F_2_ adsorption. Furthermore, the resistance change rates of sensors whose mixture ratios were 5:1, 10:1, and 20:1 showed similar values after SOF_2_ adsorption. Based on this property, we conclude that gas sensors with different mixture ratios can be utilized to better distinguish the two types of gases.

**Figure 12:**
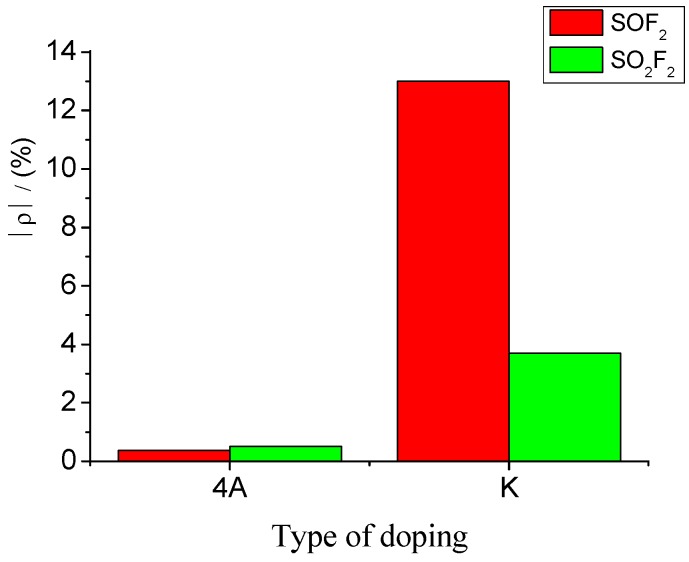
Gas-sensing selectivity of MWNT sensors to SOF_2_ and SO_2_F_2_.

Accordingly, sensors with a mixture ratio of 20:1 were selected to detect SOF_2_ and SO_2_F_2_ at 100 ppm concentration. The results were then analyzed. K-type molecular sieve is an adsorbent dedicated to SF_6_ high-voltage equipment; therefore, its ability to adsorb SF_6_ can be regarded as extremely weak, and the influence of SF_6_ as background gas can be neglected. Figure 12 shows that the resistance change rates of K-type molecular sieve-deposited sensors to SOF_2_ and SO_2_F_2_ at 100 ppm were 13% and 3.7%, respectively. Previous research has shown that 4 Å molecular sieve-deposited sensors exhibit almost no sensitivity to SOF_2_ and SO_2_F_2_ (0.37% and 0.51%, respectively). A comparison of the sensitivity of both K-type and 4 Å-type molecular sieve-deposited MWNT sensors to SOF_2_ and SO_2_F_2_ is shown in Figure 12.

The results illustrate that compared with 4 Å-type molecular sieve deposition, K-type molecular sieve deposition can obviously improve gas sensitivity of carbon nanotube sensors to SOF_2_ and SO_2_F_2_. Furthermore, sensors with a mixture ratio of 20:1 possess good selectivity towards SOF_2_; this advantage is suitable for SOF_2_ detection.

### 3.3. MWNT Sensor Response to SOF_2_ and SO_2_F_2_ at Different Concentrations 

According to the analytical results presented in Section 3.3, sensors with mixture ratios of 20:1 and 5:1 were selected to detect SOF_2_ and SO_2_F_2_ at different concentrations, respectively, so that the relationship between resistance change rate of gas sensors and gas concentration could be investigated.

**Figure 13:**
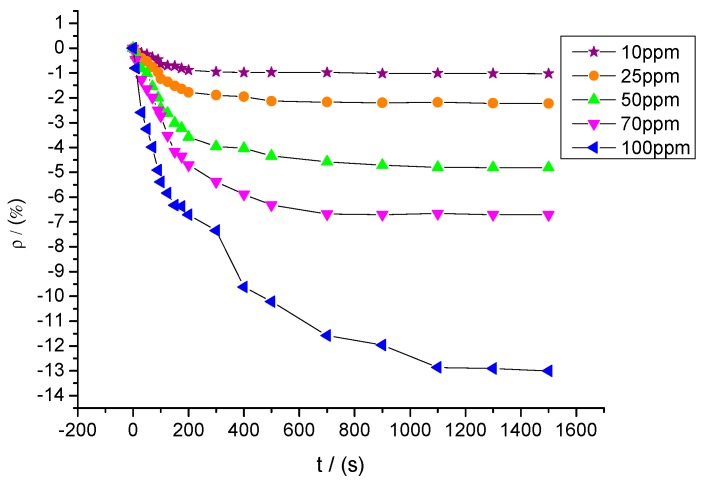
Gas-sensing response of MWNT sensors to different concentrations of SOF_2_.

Sensors with a mixture ratio of 20:1 were utilized to detect SOF_2_ at different concentrations of 10, 25, 50, 70, and 100 ppm. The response curves are shown in Figure 13. The resistance change rates of sensors to SOF_2_ at these five concentrations were approximately −1.03%, −2.23%, −4.81%, −6.71%, and −13.0%, respectively.

**Figure 14:**
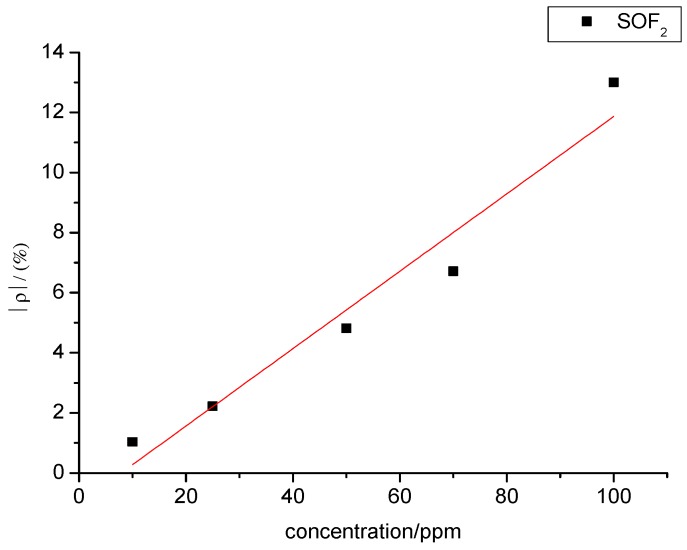
Linear relationship in the gas-sensing response of MWNT sensors to different concentrations of SOF_2_.

A certain relationship between gas concentration and change rate of sensor’s resistance can be inferred from Figure 13. The data were linearly fitted with SOF_2_ gas concentration as the independent variable and resistance change rate of the gas sensor as the dependent variable. The fitting curve is shown in Figure 14. The fitting function is y= 0.128x − 1.012, and the linear correlation coefficient (R^2^) equals 0.941.

**Figure 15:**
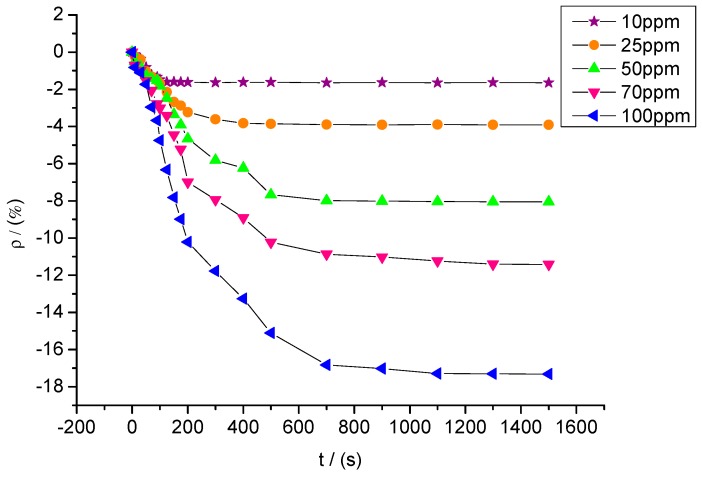
Response of MWNT sensors to different concentrations of SO_2_F_2_.

Figure 15 shows the response curves of sensors with a mixture ratio of 5:1 to SO_2_F_2_ at different concentrations of 10, 25, 50, 70, and 100 ppm. The resistance change rates of sensors to SO_2_F_2_ at these five concentrations were approximately −1.65%, −3.91%, −8.05%, −11.42%, and −17.32%.

**Figure 16:**
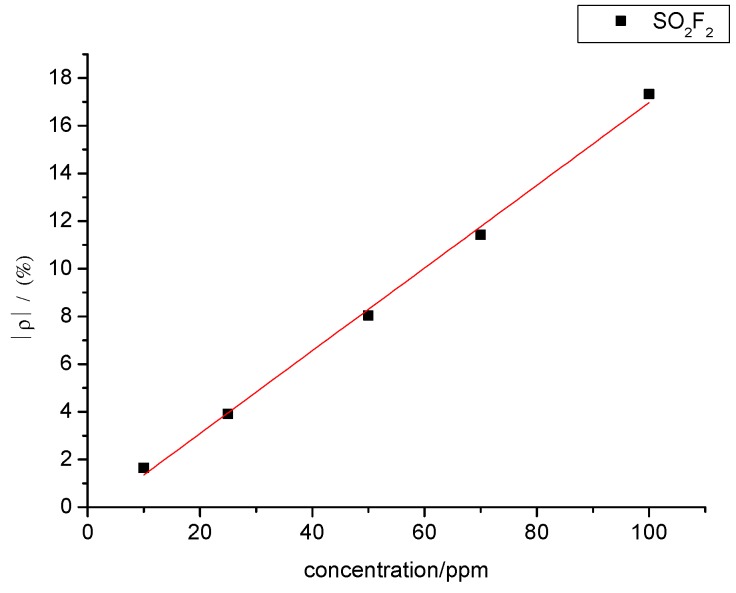
Linear relationship in the gas-sensing response of MWNT sensors to different concentrations of SO_2_F_2_.

Likewise, a certain relationship was observed between SO_2_F_2_ gas concentration and sensor resistance change rate. The experimental data were linearly fitted, and the fitting curve between SO_2_F_2_ gas concentration and resistance change rate of the gas sensors is shown in Figure 16. The fitting function is y= 0.173x − 0.378, and R^2^ equals 0.996.

Figure 14 and Figure 16 show that SOF_2_ and SO_2_F_2_ concentrations and sensor resistance change rates are linearly associated with each other when the concentration ranges from 10 ppm to 100 ppm. Therefore, gas concentration can be inferred based on the resistance change rate of the sensor.

### 3.4. Desorption and Repeatability Test for the MWNT Sensors

**Figure 17:**
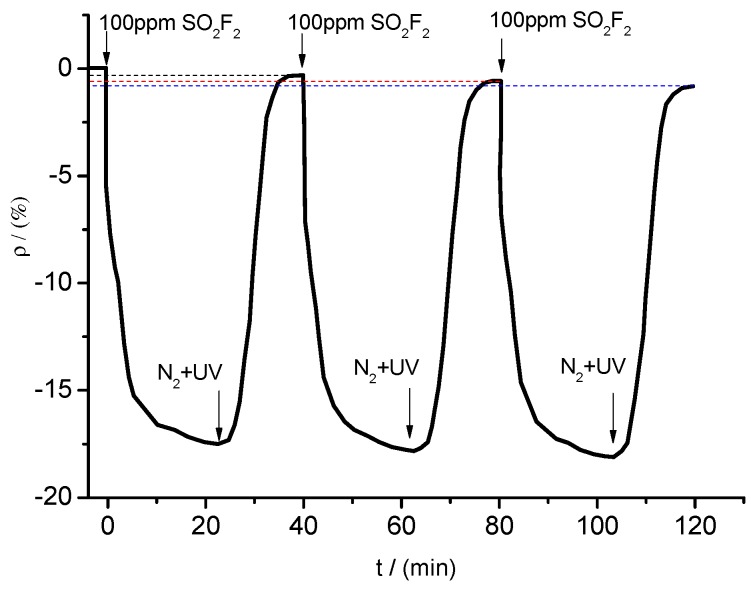
Response and recovery curves.

To investigate the stability and recovery properties of K-type molecular sieve-deposited MWNT gas sensors, the sensor with a mixture ratio of 5:1, which had been operating for five months, was selected as the research object. The sensor’s gas-sensing property was investigated through repeated experiments. The specifications of UV light exposure are a power of 8 W, wavelength of 365 nm, duration of 15 min, light intensity 3.5 mW·cm^−2^. For illustration, the sensor’s response and recovery curves to SO_2_F_2_ are on the same coordinate system. The gas sensitivity test to SO_2_F_2_ was repeated three times according to the experimental methods and procedures presented in Section 2.4. The result is shown in Figure 17.

When SO_2_F_2_ at a concentration of 100 ppm was pumped into the chamber, the resistance of the sensor changed obviously and eventually reached a stable value (fluctuating around a certain resistance value). After injection of N_2_ and UV light irradiation of the tested sensor, its resistance increased rapidly, eventually stabilizing close to its initial value. However, as the times of repeatability increased, the resistance value cannot reach exactly the initial value. The resistance change rate were in sequence of −0.302%, −0.584% and −0.819% for three times. The resistance change trends shown in Figure 17 show that desorption processes, such as N_2_ treatment and UV light irradiation, enabled the sensor’s resistance value to return to a certain value around the initial value. During the test, the resistance change trends remained similar for at least three times. Moreover, the maximum change rate remained similar and stable. Therefore, the gas sensor may be used repeatedly to detect gases with good stability and reproducibility.

### 3.5. Response Mechanism of MWNT Gas-Sensitive Sensors

As can be concluded from the above figures, the adsorption of intrinsic MWNTs to SOF_2_ and SO_2_F_2_ was very weak, which can be explained by the fact that no electron transfer occurs between intrinsic MWNTs and SOF_2_, or SO_2_F_2_. The adsorption process should be considered as physical adsorption in which van der Waals’ force plays the main part. The resistance of intrinsic MWNTs increased by approximately 2% because SOF_2_ and SO_2_F_2_ are gases possessing oxidizing capacity. As a result, the adsorption on MWNTs will restrict the electron transfer on the surface of MWNTs, causing decreasing amount of charge carrier and increase of sensor’s resistance.

**Figure 18:**
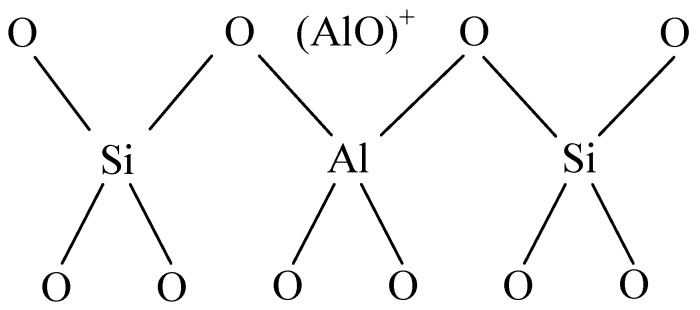
Framework structure of the acidity center.

The resistance of sensors, however, dropped after the decomposition of K-type molecular sieve, which was considered to be caused by the acidic properties due to aluminum atoms and aluminum ions from the framework and pores of the molecular sieve. Aluminum ions outside the framework can strengthen the acidity because aluminum ions which take the three-coordination break away from framework and exist in the empty space in the form of (AlO)^+^ or (AlO) _P_^+^, which can be seen in Figure 18, and thus an acidity center can be produced [24], which makes it easier for electrons to transfer from SOF_2_ and SO_2_F_2_ to the deposited MWNTs. Besides, the porous structure of K-type molecular sieve with a specific surface area ranging from 500 m^2^/g to 1000 m^2^/g, which is larger than that of MWNTs, provides more effective adsorption sites and a larger surface area. Generally speaking, increases of both surface area and adsorption sites are in favor of the permanence of gas molecular on the surface of MWNTs, thus promoting more interaction between gas molecules and the surface of the sensors.

Furthermore, the gas-sensing differences of K-type molecular sieve-deposited MWNTs to SOF_2_ and SO_2_F_2_ were also discussed. Based on the analysis above, electron transfer from gases to sensors occurs during the adsorption period. Compared with SO_2_F_2_, SOF_2_ possesses a weak oxidizing ability. That is to say, easier electron transfer from SOF_2_ to sensor may happen compared with that of SO_2_F_2_. According to the bond character of the sulfur atoms of SOF_2_ and SO_2_F_2_, the S of SOF_2_ can interact easier with C=O and C=OH groups on the surface of MWNTs introduced during the process of preparation in anhydrous ethanol. Therefore, the sensor has a stronger adsorption effect on SOF_2_ than SO_2_F_2_.

The experimental results of 4 Å-type molecular sieve-deposited MWNTs sensors showed almost no adsorption to SOF_2_ and SO_2_F_2_ while K-type molecular sieve-deposited ones showed good gas-sensing characteristics were also investigated. Preliminary interpretation was that, the main components of K-type molecular are 13X zeolite and 5 Å zeolite, which is different from 4 Å zeolite in microcosmic structures, more preciously, interconnection pattern of β cages varies, which needs to be dissected in following research.

In conclusion, compared with intrinsic MWNTs, the adsorption capacity and charge transfer ability of K-type molecular sieve-deposited MWNT sensors to SOF_2_ and SO_2_F_2_ was greatly improved. The deposited sensors performed well in terms of sensitivity and response rate. The enhancements in the sensitivity of the deposited MWNT sensors to SOF_2_ and SO_2_F_2_ also facilitated the low-concentration detection of these two gases. This finding is of great significance for the preparation of sensor arrays detecting early defects in SF_6_ gas-insulated equipment.

It should be noted that the above mechanistic discussions based on the characteristics of materials used in the experiments are still speculative, and the analysis should be considered more empirical. Therefore, a separate, detailed investigation of the sensing mechanism is essential and will be part of another manuscript. More detailed investigations are necessary for an elaborate explanation of the experimental phenomena.

## 4. Conclusions

(1) This paper focused on the detection of SF_6_ decomposition products. The 4 Å-type molecular sieve-deposited carbon nanotubes exhibited good sensitivity and selectivity to SO_2_ and H_2_S. However, these nanotubes demonstrated almost no response to SOF_2_ and SO_2_F_2_. Moreover, the gas-sensing response of intrinsic carbon nanotubes to SOF_2_ and SO_2_F_2_ were unapparent. Therefore, K-type molecular sieve-deposited carbon nanotube sensors of different mixed ratios were prepared. Gas-sensing experiments were conducted to detect two important characteristic components, namely, SOF_2_ and SO_2_F_2_.

(2) The resistance change rates of the K-type molecular sieve-deposited MWNT gas sensors to SOF_2_ and SO_2_F_2_ increased considerably. In addition, the response time was shorter compared with that of the intrinsic MWNTs. The gas concentrations of SOF_2_ and SO_2_F_2_ and the sensors’ resistance change rates exhibited a good linear relationship.

(3) The sensitivity responses of sensors deposited with different mixture ratios to SOF_2_ and SO_2_F_2_ were different from each other. Based on their sensing property, gas sensors with different mixture ratios were prepared to investigate the selectivity to the two types of gases. The adsorption capacity of sensors with mixture ratios of 5:1, 10:1, and 20:1 to 100 ppm of SOF_2_ increased significantly, whereas the adsorption capacity and response speed of MWNT sensors with a mixed ratio of 10:1 to 100 ppm of SO_2_F_2_ were enhanced.

(4) UV light was utilized to desorb residual gas molecules from the gas sensors; such light can be used to avoid chemical poisoning and prolong the sensor service time, so the sensors can be stored well and utilized repeatedly.
